# The urgency of vaccination against Covid-19 in dentists

**DOI:** 10.21142/2523-2754-0901-2021-040

**Published:** 2021-03-11

**Authors:** Luis Ernesto Arriola Guillén

**Affiliations:** 1 División de Ortodoncia, Universidad Cientifica del Sur, Lima, Perú. luchoarriola@gmail.com Universidad Científica del Sur División de Ortodoncia Universidad Cientifica del Sur Lima Peru luchoarriola@gmail.com

The requirement of vaccines for the prevention of Covid-19 has become one of the health priorities of different countries worldwide ^1^^,^^2^. However, most societies still do not have the necessary number of vaccines to cover their entire target population ^(3, 4)^. This is especially true in countries that delayed negotiations with the supply companies, and which will, unfortunately, have to wait a long time for the arrival of sufficient quantities to protect their populations. On the other hand, the global vaccination process has established priority levels among its citizens, specifically starting with the so-called first line of action that is health professionals attending Covid-19 patients, due to the great risk to which they are exposed. Additionally, the health organizations of the different countries have proposed to continue the vaccination process according to different criteria, one being the risk levels of the professions. This criterion is aimed at prioritizing professionals most exposed to contagion, but according to their daily practice, what professionals are really the most exposed to contagion?

One of the health specialties most exposed to contagion by Covid-19 is Dentistry ^5^^-^^7^, due to the evident risk of contagion by work procedures directly in the mouth of their patients, which is the main route of contagion. During the pandemic, quarantine phases were established and the practice of virtual work was encouraged. However, the different specialties of dentistry cannot be completely stopped, due to the undesired effects on the oral health of patients that would occur in ongoing treatments and the urgent and emergency dental care that are always present in daily clinical practice. In addition, virtual work in this profession is minimal and is specifically focused on specific procedures. Thus, throughout the pandemic dentists have remained active in their clinical practice with all the risk that this implies.

When considering the priority of vaccination of first line professionals, it is important to emphasize that dentists from the public and private sectors should be considered within the priority groups. This profession contributes to the maintenance of healthy oral cavity status, which has a direct impact on the general state of health and, moreover, improves the condition of the oral microflora that stimulates a favorable environment against viral and bacterial loads that are harmful to health. Finally, dentists worldwide are considered health professionals at high risk of contagion of Covid-19, and their contribution to disease prevention is great. Therefore, the necessary measures must be taken to start and maintain the process of vaccination of all dentists in Latin America.

## References

[1] Holzer F, Luna F, Manriquez T, Biller-Andorno N (2021). A matter of priority equitable access to COVID-19 vaccines. Swiss Med Wkly.

[2] Craxí L, Casuccio A, Amodio E, Restivo V (2021). Who Should Get COVID-19 Vaccine First A Survey to Evaluate Hospital Workers&apos; Opinion. Vaccines (Basel).

[3] Yu H, Yang J, Marziano V, Deng X, Guzzetta G, Zhang J, Trentini F, Cai J, Poletti P, Zheng W, Wang W, Wu Q, Zhao Z, Dong K, Zhong G, Viboud C, Merler S, Ajelli M (2021). Can a COVID-19 vaccination program guarantee the return to a pre-pandemic lifestyle. Res Sq (Preprint).

[4] Jung J (2021). Preparing for the Coronavirus Disease (COVID-19) Vaccination Evidence, Plans, and Implications. J Korean Med Sci.

[5] Siles-Garcia AA, Alzamora-Cepeda AG, Atoche-Socola KJ, Peña-Soto C, Arriola-Guillén LE (2020). Biosafety for Dental Patients During Dentistry Care After COVID-19 A Review of the Literature. Disaster Med Public Health Prep.

[6] Cabrera-Tasayco FDP, Rivera-Carhuavilca JM, Atoche-Socola KJ, Peña-Soto C, Arriola-Guillén LE (2020). Biosafety Measures at the Dental Office After the Appearance of COVID-19 A Systematic Review. Disaster Med Public Health Prep.

[7] Arellano-Cotrina JJ, Marengo-Coronel N, Atoche-Socola KJ, Peña-Soto C, Arriola-Guillén LE (2020). Effectiveness and Recommendations for the Use of Dental Masks in the Prevention of COVID-19 A Literature Review. Disaster Med Public Health Prep.

